# Self-Assembly of Optical Molecules with Supramolecular Concepts

**DOI:** 10.3390/ijms10051950

**Published:** 2009-04-27

**Authors:** Ken Okamoto, Parayalil Chithra, Gary J. Richards, Jonathan P. Hill, Katsuhiko Ariga

**Affiliations:** World Premier International (WPI) Research Center for Materials Nanoarchitectonics (MANA), National Institute for Materials Science (NIMS), 1-1 Namiki, Tsukuba, Ibaraki 305-0044, Japan

**Keywords:** Self-assembly, dye, nanostructure, surface, supramolecular chemistry

## Abstract

Fabrication of nano-sized objects is one of the most important issues in nanoscience and nanotechnology. Soft nanomaterials with flexible properties have been given much attention and can be obtained through bottom-up processing from functional molecules, where self-assembly based on supramolecular chemistry and designed assembly have become crucial processes and techniques. Among the various functional molecules, dyes have become important materials in certain areas of nanotechnology and their self-assembling behaviors have been actively researched. In this short review, we briefly introduce recent progress in self-assembly of optical molecules and dyes, based mainly on supramolecular concepts. The introduced examples are classified into four categories: self-assembly of (i) low-molecular-weight dyes and (ii) polymeric dyes and dye self-assembly (iii) in nanoscale architectures and (iv) at surfaces.

## 1. Introduction

Fabrication of nano-sized objects is one of the most important areas in nanoscience and nanotechnology. Development of top-down fabrication techniques can successfully provide well-designed micro- and nanostructures, although the hard nanomaterials obtained by top-down fabrications are not usually capable of accomplishing the more dynamic, sophisticated, and even fuzzy functions desired for nanostructured materials. Instead, soft nanomaterials with flexible properties have received much attention. These materials and related structures can be obtained through bottom-up processes from unit functional molecules, where self-assembly based on supramolecular chemistry [1,2] and designed assembly including Langmuir-Blodgett (LB) [3–5] methods and layer-by-layer (LbL) adsorption [6–10] have become crucial processes and techniques.

The preparation of self-assembled structures, although in itself academically interesting, remains a rather impractical methodology. However, formation of self-assembled structures from *functional* molecules is of great importance. Among various functional molecules, dyes have become important materials in certain areas of nanotechnology and their self-assembly behaviors have been actively studied. Although dye stuffs were previously used only for coloring, application of functional dyes to electronics, optoelectronics, and photonics has started to attract researcher’s interests in the past decade. These fields are of course deeply related with nanotechnology and nanoscience. Dyes are used as efficient absorbers of photoenergy, sometimes inducing changes of important properties. Novel functions are being pursued, including stimuli-sensitive luminescence, photoconductivity, laser sensitivity, electrification properties, dichromic properties, and non-linear optic behaviors. These unique properties are necessary for thermal devices, photography, laser technology, piezoelectric devices, and liquid crystalline devices, and are expected to be of importance in more advanced applications such as information memory and displays, energy conversion, and medical uses.

However, dyes and their properties are not simple. For example, adsorption and luminescence properties of their assembled structures are not exactly the same as those observed for single components. Spectral shifts usually occur depending highly on assembling modes such as H-aggregates and J-aggregates. They have unavoidable anisotropies within their assemblies, which are not of course observed in the solution state. Therefore, self-assembled dyes can be treated as a novel category in materials science, and researches on dye self-assemblies have been extensively carried out in recent times. In this short review, we briefly introduce recent progresses on the self-assembly of optical molecules and dyes, based mainly on supramolecular concepts. The introduced examples are classified into four categories: self-assembly of (i) low-molecular-weight dyes and (ii) polymeric dyes and dye self-assembly (iii) in nanoscale architectures and (iv) at surfaces.

## 2. Self-Assembly of Low-Molecular-Weight Dyes

**Figure 1.**
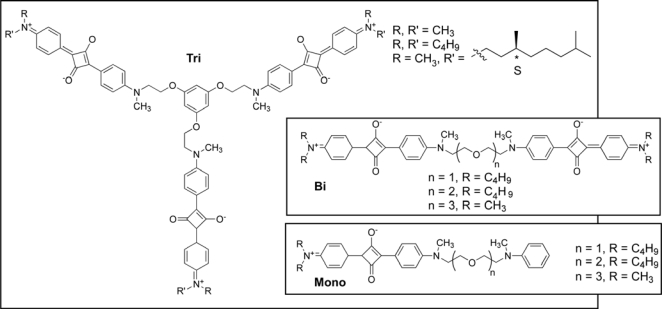
Squaraine dyes.

Mimicking the structures and the functions of biological systems are among the most challenging tasks in supramolecular chemistry. There have been several reports on the control of the overall molecular morphology and the supramolecular chirality of self-assembled architectures [11]. Ajayaghosh and co-workers prepared tripodal squaraine dyes, which form vesicular structures upon solvent evaporation of an acetonitrile solution of the dye (Figure 1) and helical architectures through the expression of molecular chirality into supramolecular helicity upon specific cation binding [12]. The images obtained by tapping-mode atomic force microscopy (AFM) of the tripodal squaraine dyes after the evaporation of the solvent from a solution of dye in acetonitrile on freshly cleaved mica sheets showed the formation of nano- to microsized spherical assemblies. Analysis of the assemblies on carbon-coated grids by transmission electron microscopy (TEM) showed the formation of relatively smaller spherical assemblies. This observation indicates that the surface influences particle formation during the evaporation of the solvent. The TEM images of tripodal squaraines reveal the vesicular nature of the spherical assemblies observed as clear contrast difference between the periphery and the inner part of the spheres. More interestingly, these hollow spherical structures of tripidal squaraine change to form nanohelices upon the addition of Ca^2+^ or Mg^2+^. This cation-controlled morphology transformation has been exploited for the design of nanohelices of the chiral tripodal squaraine dye. Circular dichroism (CD) signals and supramolecular helicity are observed only for the cation-complexed chiral dye and not for the aggregates from acetonitrile-water mixtures. This is clear example of a cation-induced morphology change of spherical assemblies into nanosized helical structures through expression of molecular chirality in a functional tripodal squaraine dye and has implications in the broad area of supramolecular dye chemistry and functional nanoarchitectures.

The effect of metal cation binding on host guest chemistry is a subject of considerable interest due to its implications in many fields such as biology, medicine and environmental studies [13]. Among various metal cation chemosensors, the design of cation-specific sensors for the selective detection of biologically important cations such as Na^+^, K^+^, Mg^2+^, Ca^2+^, and Zn^2+^ is extremely important. For this purpose, Ajayaghosh and co-workers have prepared and characterized three different squaraine tethered bichromophoric podands and an analogous monochromophore (Figure 1) [14]. The bichromophores showed high selectivity toward alkali metal ions. From the optical investigations, it is indicated that Mg^2+^ ion forms a 1:1 folded complex with the first two bichromophores whereas Ca^2+^ ions prefer to form 1:2 sandwich-shaped dimers. In contrast, the methyl-substituted bichromophore (with n = 3) forms weak 1:1 complexes with Mg^2+^, Ca^2+^ and Sr^2+^ ions. The output signals in all of these cases were confirmed by the formation of a sharp blue-shifted absorption and strong quenching of the emission of the bichromophores. The signal transduction is achieved by the exciton interaction of the face-to-face stacked squaraine chromphores in the cation complex. Interestingly, the binding behavior of the monochromophore derivative is optically silent toward Mg^2+^ and Ca^2+^. While this is clear in the case of Mg^2+^ ion, the optical silence of the monochromophore compound toward Ca^2+^ ion is rationalized on the basis of preferential formation of a ”Head-Tail-Tail-Head” structure where in which exciton coupling is not possible. The controlled supramolecular approach in combination with cation-driven exciton formation for signaling of specific cation binding is utilized in the design of cation selective chemosensors.

**Figure 2.**
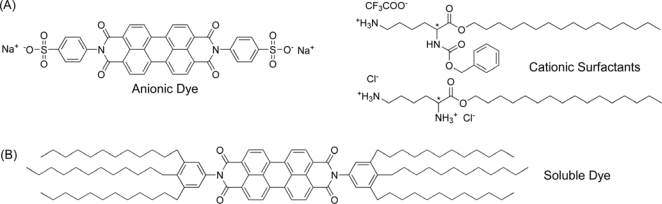
(A) Charged perylene bisimide and surfactants. (B) Perylene bisimide with alkyl tails.

The design of functional materials through self-assembly processes is becoming one of the frontiers of materials research. The objective of searching for supramolecular functional materials is that the molecular information encoded within the structure of the tectonic units induces controlled organization. To achieve this goal, ionic self-assembly is a useful interaction because the interaction can easily be tuned between charged surfactants and oppositely charged oligoelectrolytic species. By careful choice of the driving force for molecular self-assembly, Faul and co-workers have investigated the influence of chirality (from surfactants employed in the synthesis of ionic self-assembling materials) on the supramolecular organization of the resulting complexes [15]. In order to investigate the influence of chirality in perylene bisimide assemblies, a negatively charged perylene bisimide derivative and two chiral surfactants were selected (Figure 2A), and subsequently, the ionic self-assembly materials were prepared by mixing a 1wt% solution of the negative component with a solution of either cationic surfactant. CD spectroscopy shows that, upon complexation, the molecular chirality of the surfactants is expressed in the bisimide chromophore through the ionic linkage, clearly indicating that the surfactants act as structure-inducing moieties within the superstructures in both solution and the solid state. In solution, both complexes form left-handed helically stacked architectures. In the solid state, both systems are highly ordered and exhibit lamellar morphologies. The well-defined structures are the first examples of ionically self-assembled materials that show supramolecular chirality. Since it is possible to tune the material properties and characteristics of such ionically self-assembled materials through the choice of the starting materials, it is also highly possible to produce low-molecular-weight chiral liquid-crystalline materials using this strategy. This opens a wide variety of opportunities for the production of functional chiral materials for application in display technology materials. The induction of chiral effects opposite in sign to that of the starting materials offers an intriguing possibility for the production of dynamic switchable materials.

There are also growing numbers of studies on artificial functional dye assemblies possessing energy and charge transport properties [16]. Well-ordered multi-component assemblies of p- and n-type semiconducting π-electron systems have been developed for efficient charge carrier separation upon illumination [17]. Among a variety of functional dyes, perylene tetracarboxylic acid bisimides (PBIs) have been examined quite intensively due to their useful properties, such as light fastness, intense photoluminescence, and outstanding n-type semiconductivity [18]. Würthner and co-workers have prepared and characterized a novel highly soluble PBI dye (Figure 2B). The temperature and concentration-dependent NMR studies revealed formation of π-π stacked oligomeric aggregates of the dye in nonpolar solvents. Upon aggregation of the dye, a color-tunable luminescence from green to red has been observed. Furthermore, the dye possesses liquid crystalline properties. In the condensed state a hexagonal columnar liquid crystalline phase was observed. By simple solution casting, this dye afforded well-defined columnar nanostructures. X-ray diffraction and AFM studies exhibited that the columnar stacking is accomplished by rotational displacement among the molecules which is supported by theoretical calculations. Such unique π-π stacking of the dye leads to rather interesting and remarkable absorption and emission properties of the dye aggregates indicating strong electronic coupling and charge transfer interactions among the self-assembled dyes. Moreover, pulse radiolysis-time resolved microwave conductivity (PRTRMC) measurements revealed that the dye is among the best molecular semiconductors in the crystalline columnar phase. Consequently, the self-assembly, semiconducting and luminescence properties of this dye suggest such materials are good candidates for multifunctional nano-(opto)electronics.

**Figure 3.**
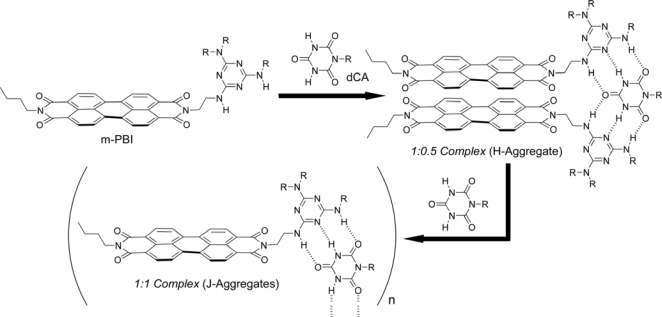
Aggregate structures of perylene bisimide upon hydrgen bonding assembly.

The local π-stacking arrangements of the chromophores greatly affect the dye’s optical and electronic properties. For instance, H-aggregated PBIs exhibit the high n-type carrier mobilities desired in electronic devices [19], the extended exciton mobility of J-aggregated PBIs could be exploited in organic solar cells. However, to date, soluble J-aggregates of core-unsubstituted PBIs with a drastic red shift of the absorption spectra are not known, despite the fact that such aggregates would certainly expand the scope of PBIs as functional materials. Yagai, Würthner and co-workers have serendipitously discovered an unprecedented phenomenon, the transformation from H-to J-aggregated perylene bisimides by using hydrogen-bond directed complexation between melamine-functionalized PBI (m-PBI) and cyanurate [20]. The H-aggregate was successfully prepared by the formation of a new supramolecular architecture consisting of ditopic cyanurate dCA and monotopic m-PBI in 1:0.5 stoichiometry (Figure 3). Further addition of dCA to the solution of m-PBI/dCA = 1:0.5 mixture, the resulting J-aggregates exhibits a remarkable red-shifted absorption band (> 100 nm), which is unprecedented for soluble core-unsubstituted perylene bisimides. Another intriguing feature of this system is the self-organization into an extended structure. When 1:1 mixtures in methylcyclohexane were kept at room temperature, a green precipitate was formed within several hours in almost quantitative yield. A striking change in absorption wavelength and the feature of spontaneous formation of a supramolecular network leads to the generation of a “green pigment” from perylene bisimide without introduction of substituents in the bay position. In the solid state as well as in solution, it has also been demonstrated that the mutual transformation between H- and J-type chromophore packing can be achieved by controlling the temperature. Based on these new findings, the construction of further functional supramolecular systems can be approached from core-unsubstituted perylene bisimides, which show optical and electronic properties that can be changed by external inputs.

## 3. Self-Assembly of Polymeric Dyes

In living cells, self-organization directs and regulates specific reactions, often at very dilute concentrations, while competing with a myriad of other possible reactants without physical barriers [21]. Nature achieves this multifarious task by using dynamic molecular recognition to sort reactive centers according to inherent molecular codes, and augmenting their effective molarity both efficiently and specifically. In other words, molecular codes in nature can translate information written in one type of molecule into another molecular language. Complex molecular codes can be created from relatively few factors such as size, shape, and charge. The interactive forces are often used simultaneously and synergistically to balance molecular rigidity and flexibility, thereby producing self-sorting effects that control reaction pathways. This concept can be regenerated by polymerization process in designed dye assemblies.

**Figure 4.**
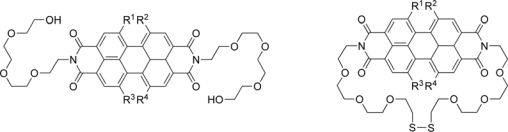
Perylene bisimide building blocks.

In order to understand dynamic self-assembly processes of molecular codes, Li and coworkers have investigated the perylene bisimides (PBIs) monomers (Figure 4) and their conjugates [22]. They have examined dynamic self-assembly between bay-substituted PBI monomers by UV/Vis and NMR spectroscopy. Monomers with different twist angles preferentially self-assemble into segregated nanostructures even in the presence of other twisted units, thus revealing their unique molecular encryption. Furthermore, to check code specificity, they compared the concentration-dependent NMR studies in a heterogeneous equimolar mixture of the monomers to those of the individual homogeneous solutions. Interestingly, the mixture self-organizes so that the monomers with different twist angles selectively self-assemble with identical species, and very little cross-assembly apparently occurs. Therefore, monomer molecular shapes act as selective molecular codes that control self-organization. To prove the molecular code concept, they employed a 1:1 heterogeneous reaction mixture of compounds, which have identical reactive functional groups and can undergo the same disulfide bond formation. If the reaction pathway was not driven by a molecular code, the reaction products would contain an indistinguishable statistical mixture of two building blocks. As expected, only the homoreaction polymer bound products, also formed in the separate homogeneous reactions, could be isolated; no cross-assembled product such as the heterogeneous ring compound was detected. These results show that a new hypothesis emerges: matching molecular size, shape, and charge produces synergistic weak intermolecular attractive forces, which impart molecular codes; such molecular codes effectively control chemical reaction pathways and products.

**Figure 5.**
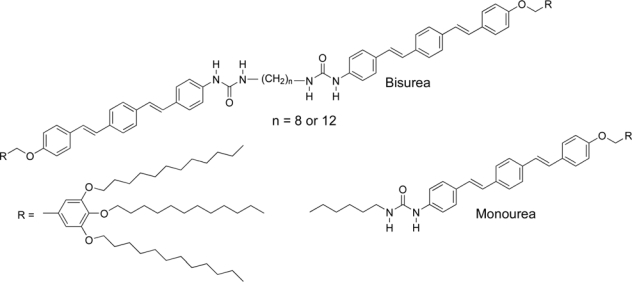
Urea-featuring oligo(p-phenylene vinylene)s.

The self-assembly of oligo(p-phenylene vinylene)s (OPVs) has been extensively studied owing to their functional applications in organic optoelectronic devices [23]. Meijer, Schenning, and co-workers put forward the concept of supramolecular electronics [24], and have investigated a new frontier of the OPV-based supramolecular polymers by using multiple hydrogen-bonding interactions. On the other hand, Ajayaghosh and coworkers have demonstrated the use of OPV organogels in photonic applications such as light-harvesting systems [25]. However, description of versatile OPV building blocks showing all of the material (such as well-defined nanoscopic fibers, organogels, or liquid crystals) morphologies are absent in the literature. For this reason, Yagai and co-workers have reported versatile self-assembling OPVs producing all the material morphologies in supramolecular polymerization and subsequent hierarchical organization [26]. In order to induce diverse material morphologies, they designed hexamethylene-tethered bisurea and monourea featuring OPV π-electronic segments and tridodecyloxyphenyl wedges (Figure 5). Bisurea exhibited the highest supramolecular polymerization ability, affording well-defined tape-like supramolecular nanofibers and fluorescent organogels in a variety of organic solvents. As demonstrated by the impaired supramolecular polymerization abilities of the bisurea and monourea, the highest supramolecular polymerization ability of the former system is a consequence of the cooperative hydrogen bonding, π–π stacking, and van der Waals interactions between the interactive moieties on both sides. Such a strong aggregation ability of bisurea in the solution phase is important for its use in nanoscopic materials or solvent-incorporated materials. By removing the solvent from gels, all the compounds can be loaded into ordered lamellar superstructures that show liquid crystallinity at moderately high temperature. A further increase in temperature results in the formation of hexagonal columnar liquid-crystalline mesophases as a result of the microsegregation of the rigid OPV parts and the molten aliphatic wedges. Thus, by changing concentration and temperature, the present OPV-ureas can produce π-electronic nanofibers, organogels, and liquid crystals with different self-organized architectures. The application of the molecular design strategy to other functional chromophores provides access to the fabrication of multiscale functional supramolecular materials.

**Figure 6.**
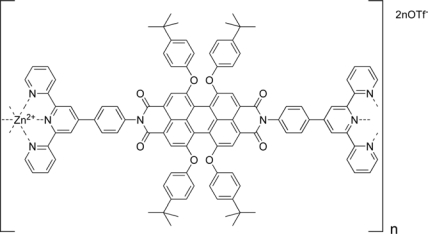
Complexation between 2,2′:6′,2″-terpyridine derivatives and Zn^2+^ ions.

Photoactive organic compounds have received much attention in the past decade as materials for organic light emitting diodes (OLEDs) and plastic solar cells. For both applications, photoactive polymers, as well as size-defined functional molecules, have been successfully developed [27]. In most cases, device fabrication involves either the spin-coating of soluble polymers or the vacuum deposition of thin films of functional molecules. Although both methods are well-established, they have certain disadvantages. Functionalized polymers are difficult to purify after the polymerization step, and defective linkages which disrupt the conjugation cannot always be avoided. Therefore, sophisticated soluble precursor polymers are necessary to overcome the obstacles. Metal-ligand coordination seems to be a good candidate for this goal. Although a large number of coordination polymer structures have been reported, only few examples are known where functional units such as dyes have been incorporated into supramolecular coordination polymers. Würthner and co-workers have investigated the complexation behavior of 2,2′:6′,2″ -terpyridine derivatives with Zn^2+^ ions, and metallo-supramolecular self-assembly of functional perylene bisimide dyes to polymeric structures (Figure 6)[28]. On the basis of the results, by Zn^2+^ complexation of monotopic and ditopic building blocks, dimer complexes and highly soluble coordination polymers have been prepared, respectively. For both dimer and polymer, reversible coordination was observed depending on the ratio of Zn^2+^/ligand, resulting for the polymer in a drastically decreased chain length when the Zn^2+^/ligand ratio exceeds 1:1. Fluorescence properties of the coordination compounds are excellent with high fluorescence quantum yields also for the polymeric compounds. The hypothesis of reversible self-assembly and de-assembly of a high-molecular-weight coordination polymer at Zn^2+^ excess was supported by the results of DOSY NMR and fluorescence anisotropy measurements. Both methods are sensitive to the diffusion coefficients of the analyte and resulted in a significantly decreased diffusion coefficient for the polymer compound in comparison to the ligand and the fragments obtained by exceeding a 1:1 ratio. Visualization of the coordination polymer was possible by AFM microscopy, and the average chain length was determined to 15 units, which is in agreement with the value of > 10 units obtained from ^1^H NMR. AFM also revealed the formation of a homogeneous monolayer on negatively charged substrates. This observation and preliminary results render this self-assembled functional system as an interesting alternative to traditional main chain perylene bisimide polymers for application in artificial light harvesting systems or organic light emitting diode fabrication.

## 4. Dye Assembly in Nanoscale Architecture

Mesoporous materials that are prepared through template synthesis of micelle supramolecular assemblies provides regular spaces in the nanometer-range [29–31] as well as microporous zeolites [32]. Mesoporous silicates as pioneered by Kuroda and coworkers [33] and Kresge *et al.* [34] are the best-known materials, and were followed by the invention of mesoporous carbon materials by Ryoo and coworkers [35]. Recently, Vinu and coworkers demonstrated syntheses of mesoporous carbon nitride [36] and mesoporous boron nitride [37]. They also invented a novel nanocarbon, the carbon nanocage [38], which can be used for effective removal of dye molecules as well as effective protein adsorption and tea component separation [39,40]. These mesoporous materials have been used for several supramolecular applications. Ariga and coworkers developed nanocomposite materials of mesoporous silica and peptides, named proteosilica [41,42], where peptide segments are densely packed in regular pores. Photochromic dye molecules such as spiropyran were co-doped into the chiral environment of these proteosilica films, resulting in an asymmetric photoreaction. Isomerization between the spiropyran form and the merocyanine form can be repeatedly induced upon alternate irradiation of visible light and UV light onto the films, respectively. Only negligible circular dichroism (CD) signals originating from the guest could be observed for the film containing the merocyanine form, but the film with the spiropyran form showed clear CD activity. Alternating irradiation with UV and visible wavelengths induced repeated reversal of these changes in the CD spectra. This system could be utilized for the development of non-destructive memory devices.

**Figure 7.**
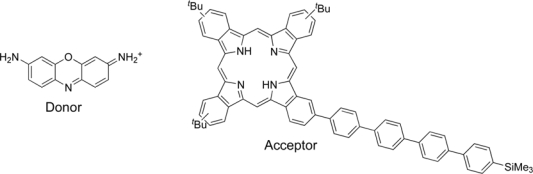
Donor and acceptor dyes trapped in zeolite L.

One dimensional channel materials are very fascinating hosts for supramolecular organization of guests species, because they are the simplest possible choice for obtaining a specific organizational pattern. Zeolite L has been shown to be a good host for the realization of such systems [43]. The size and aspect ratio of the cylindrically shaped zeolite crystals can be tuned over a wide range, adding to the extensibility of this host material. Due to the geometric constraints imposed by the one-dimensional channel structure, it is possible to afford materials with very high concentrations of monomeric dyes. In a theoretical study, Calzaferri and co-workers have examined cylinders containing energy transfer donor dye and acceptor dye molecules in such an arrangement [44]. Upon selective excitation of the donor dyes inside, the energy travels via Förster Resonance Energy Transfer (FRET) to an unexcited neighbor. After a series of steps, the electronic excitation energy reaches a luminescent trap (acceptor molecule) and is then released as fluorescence. Such artificial antenna materials are very interesting in the design of sensitized organic solar cells or fluorescent concentrators [45]. On the basis of this concept, Calzaferri, Brühwiler, Torres and co-workers have designed and prepared an artificial photonic antenna system by incorporating organic dyes into zeolite L [46]. The donor dye, shown in Figure 7, has been chosen since it has often been used for synthesizing energy transfer materials. As for the acceptor molecule, they used a stopcock shaped phthalocyanine for plugging the channel entrances of zeolite L. The donor dye was inserted into zeolite L by ion exchange from H_2_O. For the adsorption of the phthalocyanine to the channel end, an adsorption procedure from a hexane-dichloromethane suspension was suitable for the attachment of the phthalocyanine to zeolite L. UV/vis and PL spectroscopic studies show considerable energy transfer from the dye to the phthalocyanine. The combination of channel entrance functionalized zeolite L with the phthalocyanine derivatives bearing a reactive tail end group is expected to lead to more robust materials for optoelectronic applications.

**Figure 8.**
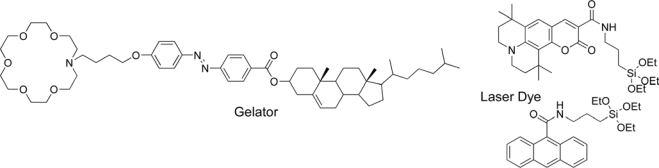
Gelator and laser dyes for luminescent silica nanotubes.

The fabrication of one-dimensional nanostructures has generated a great deal of attention in nanotechnology, because they have attractive geometries and physical properties, and numerous potential applications. Among the one-dimensional materials, silica nanotubes (SNTs) can be applied to the areas of catalysis, bioseparation, optical sensors, and nanoscale electronic devices. In the case of the organic-crystal-assisted self-assembly method, SNTs were fabricated by the addition of an ammonium hydroxide solution to an ethanol/tetraethyl orthosicate (TEOS)/tartaric acid solution. In addition, Shinkai and co-workers have employed organic gelators to prepare silica nanotubes [47]. Jang and co-workers reported silica nanotubes with novel photoluminescence (PL) characteristics which were synthesized by a sol-gel synthesis in a reverse-microemulsion system [48]. Also, Jung and co-workers have prepared novel silica nanotubes loaded with fluorescent dyes via cocondensation of modified triethoxysilyl anchor groups and TEOS in a cholesterol-based organogel system (Figure 8) [49]. They have prepared luminescent silica nanotubes (loaded with a coumarin laser dye and an anthracene laser dye) by sol-gel cocondensation of functional dyes and TEOS in a cholesterol-based organogel system. The emission colors of silica nanotubes can be tuned by using different functional dyes. Interestingly, there is a great difference in PL spectra of silica nanotubes loaded with functional dyes in ethanol solution and the solid state. In ethanol, a green light emission and a bluish green light emission of these dye-containing nanotubes were observed at 486 and 465 nm, respectively, because of the anchoring state between the dye and the nanotube. In the solid state, strong blue light emissions of these dye-containing nanotubes were observed at 482 and 483 nm, respectively. This new preparative procedure enables the utility of tunable nanometer-sized emitters for optical sensors and luminescence diodes.

## 5. Dye Assembly at Surfaces and Thin Films

Hill and coworkers have investigated multi-color porphyrins useful for versatile anion sensing [50,51] and volatile/non-volatile memory functions [52]. Molecular arrays of porphyrin molecules, fabricated by vapor deposition onto the Cu(111) surface, have also been investigated by scanning tunneling microscopy (STM) under ultra-high vacuum. A tetraphenylporphyrin derivative with *tert*-butyl groups at the *meta*-position and a hydroxyl group at the *para*-position of every phenyl ring forms a molecular array with hexagonal packing at low temperature, which shifts into a square arrangement with temperature elevation due to its conformational changes [53,54]. Very recently, ultrahigh density two-digit memory recording based on conformational difference between surface-attached porphyrin molecules was proposed [55]. Separately, coexistence of packed phases consisting of a flat conformation and a rectangle conformation was detected [56]. Surprisingly, the boundary of these two phases was filled with a single molecular line of conformation-hybrid molecules, suggesting that the molecules themselves can adjust structural discrepancy between different phases. The tetraphenylporphyrin derivative with small methyl groups on the phenyl rings allow hydrogen bond formation between them at the surface because of the lower steric hindrance of the methyl substituents [57]. In this case, formation of a Kagomé lattice was clearly confirmed by STM observation. This result suggests that appropriate design of organic molecules can lead to formation of nano-patterns within the two dimensional plane.

**Figure 9.**
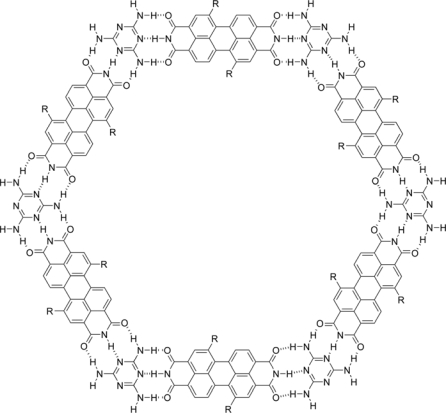
Self-assembled bimolecular network.

Champness and co-workers have recently demonstrated the formation of two-dimensional bimolecular cavities stabilized by hydrogen bonding that may be used to trap diffusing species as guest molecules [58]. Meanwhile, Stepanow and co-workers have demonstrated that the host–guest interaction may be modified through the inclusion of additional chemical groups within the framework molecules [59]. In order to put forward rational design of the constituent molecules that form the network, Champness and co-workers have explored the possibility of adding host–guest selectivity to these pores [60]. For control of the self-assembly of bimolecular networks on surfaces, they have prepared two functionalized 3,4:9,10-perylenetetracarboxylic diimide (PTCDI) derivatives, together with the melamine molecule (Figure 9).

Self-assembled structures formed by these molecules on a Ag-Si surface were studied with a scanning tunneling microscope under ultra high vacuum conditions at room temperature. Deposition of the diimide onto the Ag-Si surface results in one-dimensional molecular rows. In contrast, the self-assembly of a unimolecular hexagonal arrangement is particularly noteworthy, illustrating the stabilizing effect on introducing short-chain side groups to the PTCDI scaffold. Moreover the chemical functionalisation of the bay region of PTCDI provides a route for the rational introduction of chemically active groups on the perimeter of the pores formed in a two-dimensional supramolecular network. The modification of the unimolecular hexagonal array to the bimolecular PTCDI /melamine array, resulting in a stepwise increase in network periodicity, represents an example of dimensional control of porous-surface-based self-assembled structures through the exploitation of an additional supramolecular tecton. The contrasting entrapment behaviour observed with the di(propylthio)-PTCDI/melamine hexagonal networks relative to PTCDI/melamine and Br_2_-PTCDI/melamine systems demonstrates the potential for control, through selection of side chains, of the properties of these cavities. These results indicate a new method of functionalisation for network pores, opening up the possibility of designing nanostructured surface structures with chemical selectivity and applications in nanostructure templating.

**Figure 10.**
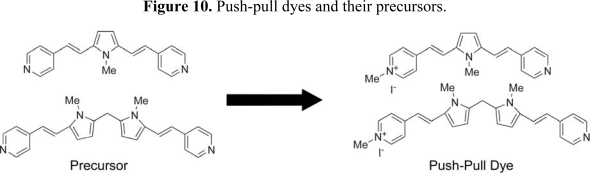
Push-pull dyes and their precursors.

In the cases of nonlinear optical (NLO) and electro-optic (EO) materials, many molecular design strategies and assembly methods have been devised to achieve polar order of the molecular building blocks on various substrates and within matrices. For example, Langmuir-Blodgett (LB) film transfer, polymer/sol-gel electric field poling, head-to-tail H-bonding, crystal engineering and molecular self-assembly are among the most preferred methods. Among the various approaches to preparing suitable electro-optic materials, layer-by-layer molecular self-assembly, and templated formation of intrinsically polar chromophores, grown directly on silicon or related substrates, is fascinating. Active second-order NLO chromophores must have an asymmetric charge distribution, however symmetrical charge distributions usually enhance third-order NLO response. The key importance of local dipole contributions to the resulting molecular hyperpolarizability of di/multibranched chromophores has been studied theoretically and experimentally.[61] Recently, Marks and Facchetti and co-workers have demonstrated that layer-by-layer self-assembled heterocycle-based dyes composed of both π-excessive (pyrrole, indole, thiophene) and pi-deficient (pyridine, diazines, quinoline, isoquinoline) heteroaromatics afford new chromophore families exhibiting both second- and third-order response.[62] The combination of a pyridinium salt and a pyrrol-2-yl substituent results in highly effective “push-pull” systems. Furthermore, they have reported the synthesis and physicochemical properties of new heterocycle-based push-pull chromophore systems (Figure 10). Chromophore precursors and the corresponding *N*-monomethyl salts undergo chemisorptive reaction with indobenzyl functionalized surfaces to afford chromophore monolayers. Spin-coating of the chromophore precursors on a benzyl-halide functionalized substrate-surface followed by a vacuum oven treatment results in films with far larger NLO responses (up to 1 order of magnitude) than films prepared by a stepwise solution-based assembly process. The second-order NLO response depends substantially on differences in film microstructures and π-electron distributions. These self-assembled chromophoric films therefore exhibit complementary nonlinear optical properties and have potential for NLO/EO applications.

## 4. Conclusions

Self-assembly is a key process in bottom-up nanotechnology. The formation of self-assembled structures from *functional* molecules is of great importance. Because dye molecules have potential functions such as photoconductivity, electrified properties, and non-linear optic behaviors, organization of these molecules becomes highly important in applications including laser technology, liquid crystalline devices, information memory and display, energy conversion, and medical uses. For development of advanced functions, the assembly and aggregation of dye stuffs in solution and/or bulk materials is of little practical use. However, organization of functional dyes within nanoscale architectures such as nanopores, surfaces, and ultrathin films is crucially important. Self-assembly in nanoscale dimensions is becoming one of the most valuable techniques in current technologies.
